# Efficacy and Safety of Bruton Tyrosine Kinase Inhibitors in Waldenström Macroglobulinemia: A Single-Center Retrospective Analysis

**DOI:** 10.7759/cureus.110182

**Published:** 2026-06-03

**Authors:** Mitali Singh, Dibakar Podder, Arjin P Jacoby, Saswata Saha, Soumyadip Chatterji, Shouriyo Ghosh, Debranjani Chattopadhyay, Jeevan Kumar, Arijit Nag, Rizwan Javed, Asish Rath, Subhosmito Chakraborty, Rakesh Demde, Pralay Ghosh, Mayur Parihar, Lateef Zameer, Gaurav Goel, Sanjay Bhattacharya, Pampi Majumder, Mammen Chandy, Reena Nair

**Affiliations:** 1 Clinical Hematology and Cellular Therapies, Tata Medical Center, Kolkata, IND; 2 Infectious Diseases, Tata Medical Center, Kolkata, IND; 3 Laboratory Hematology, Tata Medical Center, Kolkata, IND; 4 Biochemistry, Tata Medical Center, Kolkata, IND; 5 Hematopathology and Molecular Genetics, Tata Medical Center, Kolkata, IND; 6 Intensive Care, Tata Medical Center, Kolkata, IND; 7 Laboratory Hematology and Cytogenetics, Tata Medical Center, Kolkata, IND; 8 Histopathology, Tata Medical Center, Kolkata, IND; 9 Clinical Microbiology, Tata Medical Center, Kolkata, IND; 10 Microbiology, Suraksha Diagnostic Private Limited, Kolkata, IND; 11 Clinical Hematology and Cellular Therapies, Naruvi Hospital, Kolkata, IND

**Keywords:** acalabrutinib, bruton tyrosine kinase inhibitors, ibrutinib, infection, waldenström macroglobulinemia

## Abstract

Background

Waldenström macroglobulinemia (WM) is a clonal B-cell lymphoproliferative disorder characterized by the presence of monoclonal IgM paraprotein. The traditional treatment approach was chemotherapy in combination with an anti-CD20 antibody. However, the treatment approach underwent a paradigm shift with the approval of Bruton tyrosine kinase inhibitors (BTKis). This retrospective analysis aims to highlight the efficacy and safety of BTKis in WM.

Methodology

Medical records of patients with WM who received BTKis were retrospectively reviewed. Relevant details regarding demographics, clinical and laboratory investigations, imaging reports, treatment, post-treatment response, adverse events, and infection were extracted from electronic medical records. Descriptive statistics and categorical variables were presented as frequencies and percentages. Survival outcomes were estimated by the Kaplan-Meier method.

Results

In total, 22 patients received BTKis. Of these, 12 patients received ibrutinib, whereas 10 patients received acalabrutinib. The median age was 64 years. *MYD88* L265P positivity was seen in 19 (90.5%) cases, whereas *CXCR4* mutations were seen in two (13.3%) cases. The most common indication for treatment with BTKis was anemia (n = 11, 50%). Overall response rate was seen in 18 (81.8%) cases, whereas major response rates were seen in 14 (63.7%) cases. At a median follow-up of 30 months, overall survival and progression-free survival were 91% (95% confidence interval (CI) = 0.87-0.95) and 82% (95% CI = 0.77-0.87), respectively. Neutropenia and thrombocytopenia were seen in 6 (27.3%) and 7 (31.8%) cases, respectively. Bleeding, arrhythmias, and infections were seen in 2 (9.1%), 3 (13.6%), and 18 (81.8%) cases, respectively.

Conclusions

BTKis are effective in WM but are associated with high rates of hematologic and non-hematologic side effects.

## Introduction

Waldenström macroglobulinemia (WM) is a rare and indolent B-cell lymphoproliferative disorder named after Swedish physician Jan Gösta Waldenström, who described key characteristics of the disease in two patients [1,2]. The disorder is characterized by neoplastic proliferation of lymphoplasmacytic cells that infiltrates the bone marrow and the lymphoreticular system and characteristically secretes the IgM paraprotein. Patients usually present in their sixth or seventh decade with symptoms related to infiltration of the bone marrow (anemia, leukopenia, thrombocytopenia) or reticuloendothelial system (lymphadenopathy and hepatosplenomegaly), B symptoms (fever, night sweats, and significant weight loss), and IgM-related complications (hyperviscosity, cryoglobulinemia, neuropathy) [3]. There are characteristic genetic alterations seen in patients with WM. A single nucleotide change from T to C in the *MYD88* gene located on chromosome 3p22.2 is seen in up to 93% of patients, whereas *CXCR4* mutations are seen in 29% of patients [4,5]. *MYD88* is a member of the toll-like receptor (TLR) pathway that triggers downstream signalling in the presence of pathogenic activation of TLR. A mutation in *MYD88* leads to activation and auto-assembly of the myddosome that triggers the recruitment of Bruton tyrosine kinase (BTK) and interleukin-1 receptor-associated kinase (IRAK4/IRAK1), which leads to prosurvival signalling, whereas somatic mutations in *CXCR4* occur in the C-terminal region, which are similar to congenital WHIM (Warts, Hypogammaglobulinemia, Immunodeficiency, and Myelokathexis) syndrome. *CXCR4* mutations lead to robust interaction between bone marrow stromal cells and WM cells, resulting in higher disease burden and rare extramedullary involvement [4-6].

Historically, symptomatic WM has been treated with rituximab-based therapy, either as monotherapy in selected patients or in combination with chemotherapy agents such as bendamustine, cyclophosphamide, and fludarabine, or with proteasome inhibitor-based regimens (bortezomib or carfilzomib) [7-9]. The treatment landscape underwent a paradigm shift with the introduction of Bruton tyrosine kinase inhibitors (BTKis) such as ibrutinib, acalabrutinib, zanubrutinib, and pirtobrutinib [10-13]. Despite their efficacy, BTKis are associated with adverse effects, including atrial fibrillation, hypertension, bleeding related to platelet dysfunction, infections, and cytopenias [14]. There are limited studies from India reporting the efficacy and toxicities of BTKis in WM. The objectives of this study were to report the efficacy (response rates, overall survival (OS), and progression-free survival (PFS)) and safety (incidence of adverse events) of BTKis in WM.

## Materials and methods

Study design and setting

This was a single-center, retrospective, observational cohort study conducted at the Department of Clinical Hematology and Cellular Therapies, Tata Medical Center, Kolkata, a tertiary-care cancer center in eastern India. Patients treated with a BTKi between January 2019 and December 2025 were identified from the institutional electronic medical record (EMR) and were followed until death, loss to follow-up, or the data-cut date of December 31, 2025.

Eligibility criteria

Patients were eligible if they had a clinicopathologically confirmed diagnosis of WM, defined by bone marrow infiltration with lymphoplasmacytic lymphoma and a serum monoclonal IgM paraprotein, and had received at least one dose of ibrutinib or acalabrutinib in either the treatment-naïve or relapsed/refractory setting. Patients with IgM monoclonal gammopathy of undetermined significance, other B-cell lymphoproliferative disorders, or those treated exclusively with non-BTKi regimens were excluded. All eligible patients during the study period were included consecutively; no sampling was applied.

Data collection and variables

Demographic, clinical, laboratory, imaging, treatment, response, adverse event, and infection data were extracted from the EMR by the investigators using a standardized data collection form. Variables included age, sex, indication for therapy initiation, baseline serum IgM, number of prior lines of therapy, BTKi agent and duration of exposure, best response, hematologic and non-hematologic toxicities, infection episodes (site, organism, and timing), dose interruptions/reductions, progression, and survival status. Baseline risk was assessed using the International Prognostic Scoring System for WM (IPSS-WM).

Molecular testing

*MYD88* L265P and *CXCR4* mutation status were determined on bone marrow aspirate samples using allele-specific polymerase chain reaction/Sanger sequencing, as performed in the institutional molecular diagnostics laboratory. Molecular testing was performed where adequate material was available. *MYD88* was assessable in 21 patients, and *CXCR4* in 15 patients, and denominators for mutation frequencies reflect the number of patients with available results.

Response assessment

Treatment response was assessed by the treating physicians according to the response criteria of the Sixth International Workshop on Waldenström’s Macroglobulinemia (IWWM-6), based on serial serum IgM levels and clinical/radiological assessment [15]. Categories comprised complete response (CR), very good partial response (VGPR), partial response (PR), minor response (MR), stable disease (SD), and progressive disease. Overall response rate (ORR) was defined as MR or better (MR + PR + VGPR + CR) and major response (MaR) as PR or better (PR + VGPR + CR).

Toxicity assessment

Treatment-related adverse events were graded according to the National Cancer Institute Common Terminology Criteria for Adverse Events version 5.0. Infections were defined as any microbiologically and/or clinically/radiologically documented infective episode occurring during BTKi therapy and were classified by anatomical site and causative organism. Episodes without an isolated organism were recorded as clinically diagnosed infections.

Ethical considerations

The study was approved by the Institutional Review Board of Tata Medical Center (approval number: EC/WV/TMC/08/26). Given the retrospective design, the requirement for written informed consent was waived by the committee. Patient confidentiality was maintained through de-identification of all extracted data. The study conforms to the Declaration of Helsinki [16].

Statistical analysis

Data were analyzed using SPSS version 31 (IBM Corp., Armonk, NY, USA) and GraphPad Prism. Continuous variables were summarized as median (range or interquartile range) and categorical variables as frequencies and percentages. Survival outcomes, including PFS and OS, were estimated using the Kaplan-Meier method. PFS was calculated from the initiation of BTKi to progression or death. OS was calculated from the initiation of BTKi to the date of the last follow-up or death. Differences between survival distributions were evaluated using the log-rank (Mantel-Cox) test. A two-sided p-value <0.05 was considered statistically significant.

## Results

In total, 22 patients were included in the current analysis. The median age was 64 years, and males were commonly affected (77.3%). The median IgM was 3.1 g/dL (0.37-10.7 g/dL). *MYD88* L265P mutation was detected in 19 of 21 tested patients (90.5%), while *CXCR4* mutations were identified in 2 of 15 tested patients (13.3%). Risk stratifications were performed using IPSS-WM at baseline in 15 cases, and 10 (66.7%) patients were high risk. The most common indication for treatment with BTKis was anemia (n = 11, 50%), followed by hyperviscosity (n = 7, 31.8%), lymphadenopathy (n = 3, 13.7%), cytopenia (n = 3, 13.7%), splenomegaly (n = 1, 4.5%), and neuropathy (n = 1, 4.5%). The median prior line of therapy received was two (0-4). Overall, 12 (54.5%) patients received ibrutinib, while 10 (45.5%) patients were given acalabrutinib (Table 1).

**Table 1:** Baseline characteristics, treatment received, and response rates. Percentages for *MYD88*, *CXCR4*, and IPSS-WM are calculated from the number of patients with available data. BTKi: Bruton tyrosine kinase inhibitor; IgM: immunoglobulin M; IPSS-WM: International Prognostic Scoring System for Waldenström macroglobulinemia; IQR: interquartile range

Characteristic	N = 22 (%)
Age in years, median (range)	64 (53–83)
Gender	
Male	17 (77.3%)
Female	5 (22.7%)
Indications for therapy initiation	
Symptomatic anemia	11 (50%)
Hyperviscosity	7 (31.8%)
Lymphadenopathy	3 (13.7%)
Cytopenia	3 (13.7%)
Splenomegaly	1 (4.5%)
Neuropathy	1 (4.5%)
IgM prior to BTKi (g/dL), median (range)	3.1 (0.37–10.7)
Molecular genetics	
MYD88 (n = 21)	19 (90.5%)
CXCR4 (n = 15)	2 (13.3%)
IPSS-WM (n = 15)	
Low	2 (13.3%)
Low intermediate	1 (6.7%)
Intermediate	2 (13.3%)
High	10 (66.7%)
Lines of therapy, median (range)	2 (0–4)
BTKi	
Ibrutinib	12 (54.5%)
Acalabrutinib	10 (45.5%)
Duration of BTKi exposure (months), median (IQR)	8.66 (4.8–46.8)
Response rates (best response)	
Complete response (CR)	2 (9%)
Very good partial response (VGPR)	3 (13.6%)
Partial response (PR)	9 (41%)
Minor response (MR)	4 (18.2%)
Stable disease (SD)	4 (18.2%)
Major response (PR + VGPR + CR)	14 (63.7%)
Overall response (MR + PR + VGPR + CR)	18 (81.8%)

**Figure 1:**
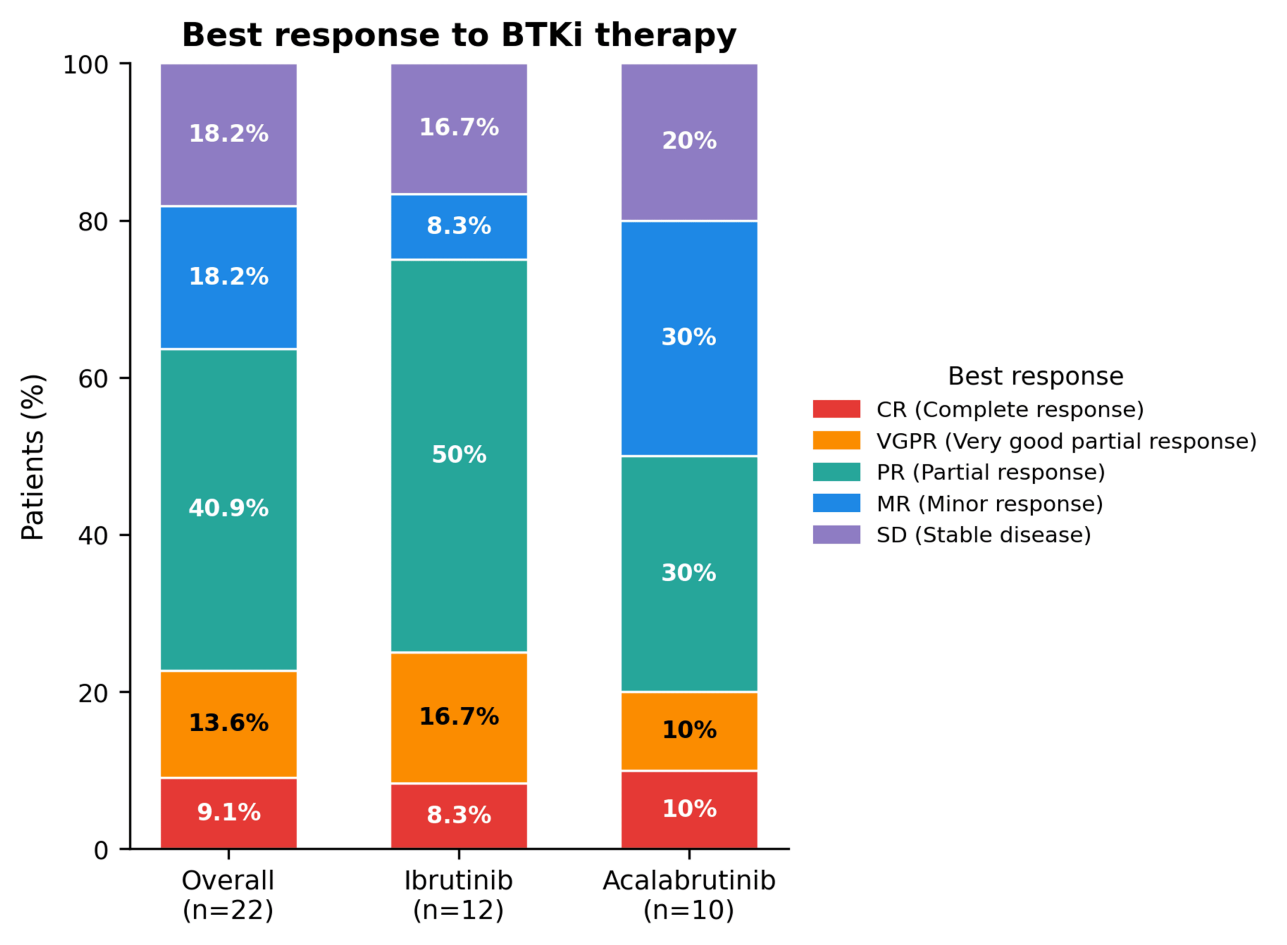
Response rate in the overall cohort, ibrutinib, and acalabrutinib. Best response of ibrutinib, acalabrutinib, and the entire cohort in Waldenström macroglobulinemia. ORR and MaR with ibrutinib were 83.3% and 75%, respectively, whereas ORR and MaR with acalabrutinib were 80% and 50%, respectively. BTKi: Bruton tyrosine kinase inhibitor; CR: complete response; VGPR: very good partial response; PR: partial response; MR: minor response; SD: stable disease; MaR: major response; ORR: overall response

In the entire cohort, CR was seen in two (9%) patients, VGPR was seen in three (13.6%) patients, PR was seen in nine (41%) patients, MR was seen in four (18.2%) patients, and SD was seen in four (18.2%) patients. ORR was seen in 18 (81.8%) cases, whereas MaR rates were seen in 14 (63.7%) cases. In the ibrutinib cohort, best responses were as follows: CR (n = 1, 8.3%), VGPR (n = 2, 16.7%), PR (n = 6, 50%), MR (n = 1, 8.3%), SD (n = 2, 16.7%), ORR (n = 10, 83.3%), and MaR (n = 9, 75%) whereas in the acalabrutinib cohort CR, VGPR, PR, MR, SD, ORR and MaR were seen in one (10%), one (10%), three (30%), three (30%), two (20%), eight, (80%) and five (50%) cases respectively (Figure 1). No patient had primary refractory disease, but three patients (13.6%) progressed in the entire cohort after initial response (Table 1).

**Figure 2:**
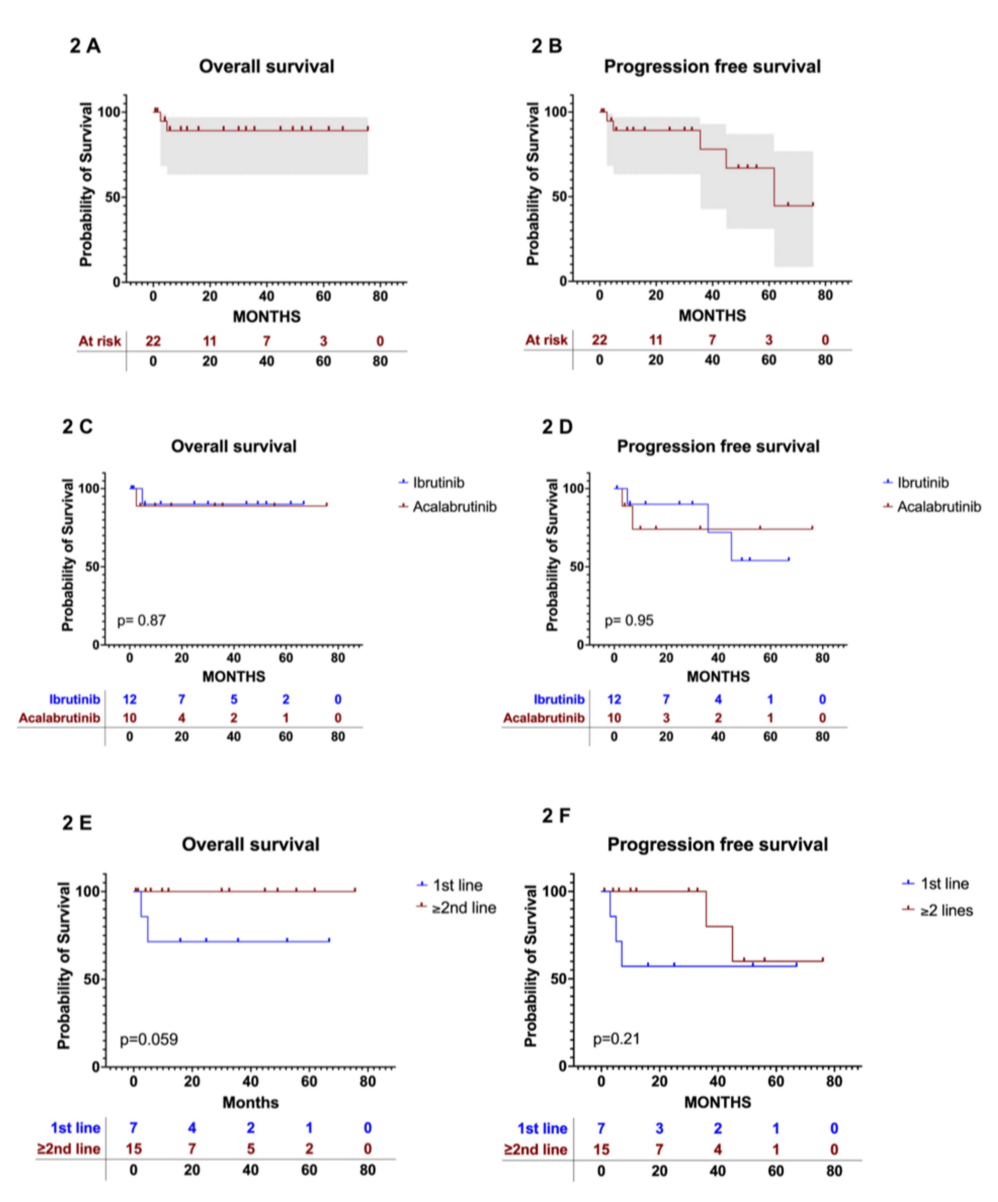
Kaplan Meier survival curves for overall survival, progression free survival, and log-rank test. (A) Overall survival (OS) of the entire cohort. Median OS: not reached. Two- and five-year estimated OS are 91% (95% CI = 0.87–0.95) and 89% (95% CI: 0.84–0.93), respectively. (B) Median progression-free survival (PFS) was not reached. Two- and five-year PFS are 82% (95% CI = 0.77–0.87) and 62% (95% CI = 0.52–0.68), respectively. (C) OS of ibrutinib and acalabrutinib cohort. No statistical significance between ibrutinib and acalabrutinib (p = 0.87). (D) PFS of ibrutnib and acalabrutinib. No statistical significance between ibrutinib and acalabrutinib. Log=rank p-value = 0.95. (E) Kaplan-Meier plot with log-rank test plots show no statististical difference on overall survival on use of Bruton tyrosine kinase inhibitor (BTKi) as first lines and ≥second lines (p = 0.059). (F) PFS show no statistical significance stratified by use of BTKi as first line and ≥second line (p = 0.95).

**Figure 3:**
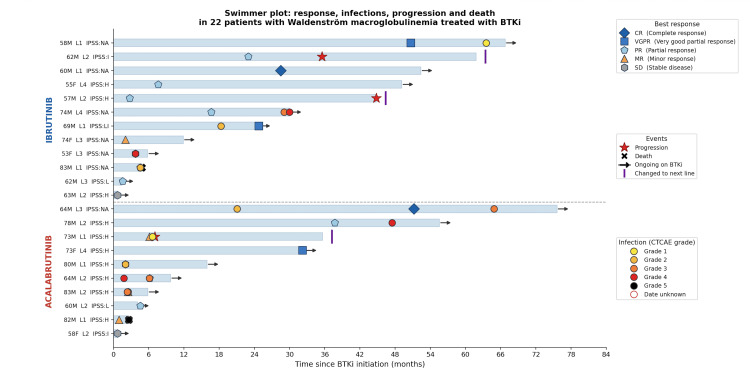
Swimmer’s plot of the entire cohort depicting response, infection, progression, and death. Each lane represents one patient (sorted within drug groups by length of follow-up). Lane label: age + sex, L = line of BTKi, IPSS-WM (H = high, I = intermediate, L= low, LI = low-intermediate, NA= not assessed).

Median OS was not reached at a median follow-up of 30 months. Estimated two- and five-year OS were 91% (95% confidence interval (CI) = 0.87-0.95) and 89% (95% CI = 0.84-0.93), respectively. Within the cohort of 22 patients, two deaths occurred, both secondary to septic shock and pneumonia. Median PFS was not reached. The estimated two- and five-year PFS were 82% (95% CI = 0.77-0.87) and 62% (95% CI = 0.52-0.68), respectively (Figures 2A, 2B). There was no significant difference in OS and PFS between ibrutinib and acalabrutinib (Figures 2C, 2D). There was no significant difference in OS and PFS if a BTKi is used as a first line or in subsequent lines (Figures 2E, 2F). Swimmer’s plot (Figure 3) highlights response, infection, progression, and death of the entire cohort.

Among hematological toxicities, neutropenia and thrombocytopenia were seen in 27.3% and 31.8% of cases, respectively. Bleeding, arrhythmias, and infections were seen in 9.1%, 13.6%, and 81.8% of cases, respectively. Drug-wise distribution of toxicities, interruption, and stoppage is summarized in Table 2. Bacterial infections (38.9%) were more commonly seen compared to viral (22.2%) and fungal infections (5.5%). Two cases of mycobacterial infections were also seen. Characteristics and site of infections are highlighted in Table 3.

**Table 2:** Toxicity profile, drug interruption, and dose reduction of the overall cohort, ibrutinib, and acalabrutinib. Toxicities graded per CTCAE; values shown are Grade 2–4 events. Percentages are calculated within each column. BTKi: Bruton tyrosine kinase inhibitor; CTCAE: Common Terminology Criteria for Adverse Events

Adverse event (Grade 2–4)	Overall cohort (N = 22)	Ibrutinib (N = 12)	Acalabrutinib (N = 10)
Neutropenia	6 (27.3%)	3 (25%)	3 (30%)
Thrombocytopenia	7 (31.8%)	4 (33.3%)	3 (30%)
Bleeding	2 (9.1%)	1 (8.3%)	1 (10%)
Arrhythmias	3 (13.6%)	2 (16.7%)	1 (10%)
Hypertension	2 (9.1%)	1 (8.3%)	1 (10%)
Infections	18 (81.8%)	8 (66.7%)	10 (100%)
Drug interruption	14 (63.7%)	9 (75%)	5 (50%)
Dose reduction	4 (18.2%)	3 (25%)	1 (10%)

**Table 3:** Infection characteristics. Percentages are calculated from the 18 patients who developed an infection; some patients had infection at more than one site or with more than one pathogen. CNS: central nervous system; CMV: cytomegalovirus; RSV: respiratory syncytial virus

Infection characteristic	N = 18 (%)
Site of infection	
Respiratory	5 (27.8%)
Bloodstream infection	4 (22.2%)
Urinary	3 (16.8%)
CNS	2 (11.1%)
Gastrointestinal	1 (5.5%)
Lymph node	1 (5.5%)
Skin and soft tissue	1 (5.5%)
Bone	1 (5.5%)
Pathogen	
Virus	
Rhinovirus/Enterovirus	1 (5.5%)
Parainfluenza	1 (5.5%)
RSV	1 (5.5%)
CMV	1 (5.5%)
Bacteria	
Mycobacterium tuberculosis	2 (11.1%)
*Nocardia* sp.	1 (5.5%)
Klebsiella pneumoniae	1 (5.5%)
*Enterobacter* sp.	1 (5.5%)
*Shigella* sp.	1 (5.5%)
*Janibacter* sp.	1 (5.5%)
Fungal (*Aspergillus*)	1 (5.5%)
No organism isolated	6 (33.3%)

## Discussion

Although the use of BTKis in various B-cell malignancies is common, due to the rarity of WM, there is a paucity of data on the use of BTKis in WM from India. This study highlights the efficacy and the challenges of using ibrutinib and acalabrutinib in WM. We included 22 patients with WM with a median age of 64 years. The current cohort had 66.7% of high-risk patients as per IPSS-WM, 90.5% of patients had a *MYD88* L265P mutation, and 68.2% received at least one prior line of therapy.

The pivotal trial by Treon et al. included a heavily pretreated cohort of WM and showed an ORR and MaR of 90.5% and 73%, respectively, while OS and PFS at two years were 95.2% and 69.1%, respectively, which is similar to the present study [10]. The updated analysis of this study showed an ORR and MaR of 90.5% and 79.4%, respectively, whereas five-year PFS and OS were 54% and 87%, respectively [17]. There are limited studies of acalabrutinib in WM. Owen et al. used single-agent acalabrutinib in WM; 86.8% of patients in their cohort had received prior treatment, and the ORR and MaR were 93% and 78%, respectively. This is high when compared to the current study, where ORR and MaR were 80% and 50%, respectively. Several factors may explain the somewhat lower MaR rate seen in our cohort compared with the pivotal acalabrutinib and ibrutinib studies. Our patients had received a median of two prior lines of therapy (range = 0-4), and 66.7% were classified as high‐risk by IPSS‐WM, which is more pretreated and higher risk than the populations in many published trials. In addition, response in WM is known to be a genotype‐dependent phenomenon: while *MYD88* L265P‐mutated, *CXCR4* wild‐type patients show the deepest and most rapid responses to covalent BTKi, those harboring *CXCR4* nonsense mutations, *MYD88* wild‐type disease, or *TP53* alterations exhibit slower onset of response, lower MaR rates, and shorter PFS. The final analysis of the ASPEN study and other comparative data suggest that zanubrutinib may partly overcome these adverse molecular determinants in patients with *CXCR4*‐mutated or *MYD88* wild‐type disease [11]. Although *CXCR4* testing was performed in only 15 of our 22 patients and *TP53* testing was not done, two patients in our cohort progressed despite a documented initial response, raising the possibility of an underlying adverse genotype that was not fully characterized. In a recent multicenter real‐world Israeli cohort of zanubrutinib in predominantly relapsed/refractory and high‐risk WM patients, the ORR was 83% with 18‐month PFS of 60.5%, broadly comparable to our observations and reinforcing that real‐world outcomes with BTKis tend to lag behind those reported in carefully selected trial populations [18].

Ibrutinib was associated with hematologic and non-hematologic toxicities owing to its off-target inhibition of various kinases. Grade 2-4 neutropenia and thrombocytopenia were seen in 25% and 33% of patients, respectively, in our ibrutinib cohort. Although the rates of neutropenia were similar, the incidence of thrombocytopenia was lower in the studies by Treon et al., Trotman et al., and Dimopoulos et al. [10,11,19]. The incidence of atrial fibrillation, hypertension, and bleeding was similar to other reported studies [10,17,19]. Acalabrutinib was also associated with similar hematologic and non-hematologic toxicities as per other studies in WM [12,20].

Similar rates of infection were also seen with Ibrutinib in studies by Treon et al. and Tilly et al. [10,21]. Although there is limited data on infection risk with the use of acalabrutinib in WM, rates of infections were similar in cohorts of chronic lymphocytic leukemias [22]. When compared with the bendamustine-rituximab (BR) regimen, BTKi use is associated with higher rates of infection in WM patients [23-25]. There is limited data in WM about the site and nature of pathogens. The ELEVATE-RR trial showed a higher incidence of respiratory tract infections (47.4%), followed by pneumonia (21.8%), urinary tract infection (9.4%), and sepsis (4.5%) in the ibrutinib arm, whereas acalabrutinib-treated patients had slightly lower rates of infections, with respiratory tract infections (41.8%), pneumonia (18.3%), urinary tract infections (16.7%), and sepsis (4.6%). Both arms showed a 4-5% incidence of opportunistic infections [22]. Gilbert et al. found higher rates of fungal infections with the use of BTKis in Chronic lymphocytic leukemia and other B-cell non-Hodgkin lymphoma patients [26]. The overall infection rate of 81.8% observed in our cohort, with notable opportunistic pathogens, including *Aspergillus*, *Nocardia*, cytomegalovirus, and two cases of *Mycobacterium tuberculosis*, is a striking finding and warrants particular attention. Although BTK inhibition is not classically associated with deep neutropenia, BTK has critical off‐target roles in macrophage phagocytosis, neutrophil chemotaxis, and innate immune signalling, and prolonged therapy is frequently complicated by hypogammaglobulinaemia, all of which collectively predispose patients to opportunistic infection. Prior use of chemo-immunotherapy and proteasome inhibitors also predisposes to high rates of infection. In a more recent French multicenter study of ibrutinib‐treated patients admitted to intensive care for severe infection, invasive fungal disease accounted for almost one‐fifth of presentations, and viral and fungal infections were significantly more common compared with chemotherapy‐treated controls; day‐90 mortality reached 55% [27]. These observations underline the need for a high index of clinical suspicion and a low threshold for advanced diagnostic workup in BTKi‐treated WM patients who develop respiratory or neurological symptoms. The occurrence of two cases of active tuberculosis in our cohort further highlights the additional infection burden faced by BTKi‐treated patients in tuberculosis‐endemic regions such as India, where pre‐treatment screening for latent tuberculosis and a structured approach to anti‐viral, anti‐pneumocystis, and immunoglobulin replacement strategies, individualized to patient risk, deserve prospective evaluation.

Although newer-generation BTKis, such as zanubrutinib and pirtobrutinib, have a safer toxicity profile compared to ibrutinib and acalabrutinib, in the current treatment landscape in India, zanubrutinib is expensive while pirtobrutinib is not available for use [11,13]. To our knowledge, this is the first study from India to report the experience of BTKis in the treatment of WM.

The current study has a few limitations. Its retrospective design, small sample size, and single-center design limited generalizability. Incomplete CXCR4 testing limits the ability to perform meaningful subgroup analysis incorporating molecular genetics and heterogeneous prior lines of therapy. A short median follow-up period resulted in undercapturing of late progression and toxicities.

## Conclusions

BTKis are associated with high response rates in WM, but due to off-target toxicities such as hematological, arrhythmias, and immune dysfunction, there should be awareness among physicians, patients, and caregivers about regular follow-up and close monitoring.
